# Draft Genome Sequence of *Lactobacillus plantarum* Strain Lp91, a Promising Indian Probiotic Isolate of Human Gut Origin

**DOI:** 10.1128/genomeA.00976-13

**Published:** 2013-11-21

**Authors:** Sunita Grover, Vineet K. Sharma, Rashmi H. Mallapa, Virender K. Batish

**Affiliations:** Molecular Biology Unit, Department of Dairy Microbiology, National Dairy Research Institute, Karnal, Haryana, Indiaa; Metagenomics and Systems Biology Laboratory, Indian Institute of Science Education and Research (IISER), Bhopal, Indiab

## Abstract

*Lactobacillus plantarum* is a highly versatile species among lactic acid bacteria that has been widely isolated from highly diversified ecological niches, including the gastrointestinal tract. Here, we report the first draft genome sequence of an Indian isolate of the probiotic strain *L. plantarum* Lp91, isolated from human gut.

## GENOME ANNOUNCEMENT

*Lactobacillus plantarum* merits special attention in view of its multitude of bioactive metabolic functions beneficial for human health and its presence in many environmental niches, including dairy and meat products and a variety of vegetable fermentations, in addition to the human gastrointestinal (GI) tract (1). The complete 3.3-Mb genome sequence of *L. plantarum* WCFS1 (from saliva) has been determined (2) and resequenced and completely annotated recently by Siezen et al. (3). In addition, the complete genomes of three other *L. plantarum* strains have also been sequenced, *viz*. *L. plantarum* ST-III (4), *L. plantarum* JDM1 (5), and *L. plantarum* strain NC8 (CCUG 61730) (6).

*L. plantarum* strain Lp91 is an indigenous isolate of Indian gut origin and has been identified by 16S rRNA (GQ922598) sequencing as well as housekeeping genes, *viz*. *pheS* (KC509913.1), *tkt4* (KC509921.1), *pgm* (KC509919.1), *gyr* (KC509917.1), and *purK1* (KC509920.1). Lp91 has expressed several probiotic and functional attributes, such as high acid and bile tolerance, colonization potential, cholesterol assimilation, reduction in low-density lipoprotein (LDL)-cholesterol, and antibacterial, antioxidative, anti-inflammatory, and immunomodulatory potentials in both *in vitro* cell lines (THP-1 and HT-29) and *in vivo* animal models (7–15). Since *L. plantarum* Lp91 serves as a candidate probiotic for developing probiotic fermented dairy products and powders and sachets, its whole-genome sequence was deciphered.

Genome sequencing was performed using an Illumina (HiScanSQ) genome analyzer. A total of 13,098,338 high-quality reads were obtained after filtering, with a Phred score of <20, and were used for alignment with *L. plantarum* WCFS1 (LpWCFS1), which was used as the reference genome. As much as 80.63% of the total reads were aligned with the reference genome, with 89.11% genome coverage and a total gap length of 0.36 Mb. A total of 15,820 single nucleotide polymorphisms (SNPs) were found by genomic comparison of Lp91 with LpWCFS1.

The draft genome of Lp91 was assembled into a single circular chromosome of 3,308,256 bp by use of the reference LpWCFS1genomic sequence and the Burrows-Wheeler Aligner (BWA) (16), by inserting “N” to fill the gaps. The G+C content of Lp91 was 45%, and 2,779 predicted protein-coding genes were identified by using Glimmer 3.02 (17) followed by manual curation. As many as 2,346 (84.4%) genes were annotated with known functions by use of BLASTP against NCBI nonreduntant (NR) and Clusters of Orthologous Groups (COG) databases (18). However, 433 (15.6%) genes were annotated as hypothetical, conserved hypothetical, or unnamed protein products. The Lp91 strain includes five rRNA gene operons and 65 tRNA genes in the genome. In addition, 1,586 genes were classified into 20 COG functional classes, of which the most abundant classes were transcription (16%) and carbohydrate transport and metabolism (13%).

### Nucleotide sequence accession numbers.

This whole-genome shotgun project has been deposited at DDBJ/EMBL/GenBank under the accession number AXDQ00000000. The version described in this paper is version AXDQ01000000.
